# Temporal Changes of Leaf Spectral Properties and Rapid Chlorophyll—A Fluorescence under Natural Cold Stress in Rice Seedlings

**DOI:** 10.3390/plants12132415

**Published:** 2023-06-22

**Authors:** Árpád Székely, Tímea Szalóki, Mihály Jancsó, János Pauk, Csaba Lantos

**Affiliations:** 1 Research Centre for Irrigation and Water Management, Institute of Environmental Sciences, Hungarian University of Agriculture and Life Sciences, Anna-Liget Str. 35, H-5540 Szarvas, Hungary; szaloki.timea.palma@uni-mate.hu (T.S.); jancso.mihaly@uni-mate.hu (M.J.); 2 Cereal Research Non-Profit Company, H-6726 Szeged, Hungary; janos.pauk@gabonakutato.hu

**Keywords:** absorbance, chilling stress, reflectance, spectral indices, photosynthetic quenching analysis

## Abstract

Nowadays, hyperspectral remote sensing data are widely used in nutrient management, crop yield forecasting and stress monitoring. These data can be acquired with satellites, drones and handheld spectrometers. In this research, handheld spectrometer data were validated by chlorophyll-a fluorescence measurements under natural cold stress. The performance of 16 rice cultivars with different origins and tolerances was monitored in the seedling stage. The studies were carried out under field conditions across two seasons to simulate different temperature regimes. Twenty-four spectral indices and eleven rapid chlorophyll-a fluorescence parameters were compared with albino plants. We identified which wavelengths are affected by low temperatures. Furthermore, the differences between genotypes were characterized by certain well-known and two newly developed (AAR and RAR) indices based on the spectral difference between the genotype and albino plant. The absorbance, reflectance and transmittance differences from the control are suitable for the discrimination of tolerant-sensitive varieties, especially based on their shape, peak and shifting distance. The following wavelengths are capable of determining the tolerant varieties, namely 548–553 nm, 667–670 nm, 687–688 nm and 800–950 nm in case of absorbance; above 700 nm for reflectance; and the whole spectrum (400–1100 nm) for transmittance.

## 1. Introduction

Precision agriculture is a key component of sustainable agricultural systems, which uses advanced information, communication and data analysis techniques. Remote sensing is one of the possible ways to produce deep analyses about the current state of a plant in a non-destructive way. A lot of spectral data are typically produced by satellite [1,2,3], UAV [4,5,6] or handheld spectrometers [7]. The measurement of reflectance using portable spectral measurement instruments is the most advantageous method of data acquisition [8].

The red edge spectral region, which is between 670 and 800 nm, is by far the most thoroughly researched region of the plant spectrum [9]. This might be because bands in the center of the absorption feature are less susceptible to changes in chlorophyll content than in the near-infrared region (690–730 nm) [10]. Moreover, several chemical and physical traits of the plant affect the reflection of solar energy [11]. In addition to carotenoids [12], anthocyanins and flavonols [13], the trichomes and thickness [14] of the leaves also affect the spectrum.

The chlorophyll shows two maximum absorption peaks at 450–500 nm and 640–700 nm (for the 479–492 nm and 673 nm bands, respectively) under normal circumstances [15]. In the visible bands, the typical reflectance range is just 10–20%, while in the near-infrared area at 700–1000 nm it is 40–50%. When the environmental stress is onset, the spectral line shape is changing [16]. These changes can be well described by a wide range of vegetation and difference indices. The difference in absorbance between two wavelengths (670 and 720 nm) is known as the absorbance difference index (IAD), and it has a strong correlation with the chlorophyll-a concentration [17]. The nitrogen content of rice is correlated with the difference spectral index (DSI) [18]. There are also stressed and non-stressed difference indices [10,16,19] and temporal difference indices, such as between the stage of pre-flowering and ripening [5]. In addition to these, there are numerous vegetation indices that relate to the stress response. The phytochemical reflectance index (PRI) and Carter index (CTR 1 and CTR 2) are known as the general stress response indices [10,20,21]. The normalized difference red edge index (NDRE) can be used to detect relatively late-stage heat stress [22] and it also has a strong correlation with the levels of calcium and magnesium in wheat genotypes grown on sodic soils [23]. Numerous plant stressors, beside nitrogen stress, can cause red edge shifts [24]. When the leaves are in the growing phase, the red edge inflection point (REIP) shifts toward longer wavelengths, then shifts back to shorter wavelengths when there is a water shortage and discoloration of the leaves [25]. However, when plants are exposed to heavy metal pollution, the inflection point increases [26]. The effectiveness of these indices is usually verified by photosynthetic parameters. In experimental plant biology, a rapid chlorophyll-a fluorescence analysis (JIP test) is frequently used to gauge how the photosynthetic system would react to various conditions. The JIP test provides a lot of parameters for analyzing adaptation mechanisms to different types of stress [27]. The JIP test can be used not only to assess different stress types but also to distinguish specific responses to a particular stress type; for example, because of genotypic differences, differences in stress intensity or differences in the developmental stage of the stressed plants [28]. The O–J part of the fluorescence increase relates to the closure of the PSII reaction centers due to the reduction in QA. The J–I part of the curve corresponds to the plastoquinone (PQ) pool. The I–P part of the induction curve is typically attributed to the reduction in electron transporters of the PSI acceptor side [27,29]. Among the spectral indices, the best documented parameter that is closely related to photosynthetic efficiency is the PRI [30]. Based on the observation of Sukhova et al., the PRI correlates with non-photochemical energy (NPQ) [21]. Since it is derived from narrow-band reflectance at 531 and 570 nm, it is correlated with the carotenoid content and connected to xanthophyll-de-epoxidation-mediated heat dissipation [30]. In addition, the PRI correlates well with the Fv/Fm ratio [31], PSII radiation use efficiency and CO_2_ uptake [32]. Other indices, such as the NDRE [31], NDVI and SIPI (structure-insensitive pigment index) are also correlated with the parameters of photosynthesis (Fo, Fm, Fv/Fm, Fv/Fo, NPQ) [33].

The chilling temperature seriously affects the growth and yield in rice. Rice is relatively sensitive to temperatures below 15 °C, which causes different effects in different growth stages, such as the germination, seedling, vegetative and reproductive stages [34,35]. Photosynthetic activity is severely hampered by cold stress, which also harms photosynthetic pigments [36]. Leaf chlorosis, a common symptom of chilling stress, is caused by the inhibition of the synthesis of chlorophyll and destruction of existing chlorophyll [37]. Moreover, as the nitrogen uptake slows down [38], the leaf color changes even more dramatically. Low temperatures can cause the plastoquinone levels to drop and PSII reaction centers to sustain photoinhibitory degradation. In addition, a physiological difference between rice subclasses has been reported. *Japonica* rice generally has thick and small leaves with relatively low stomatal density, which facilitates the maintenance of water balance at low temperatures [39,40]. Furthermore, the quantum photochemistry yields are generally lower and the NPQ is higher in *japonica* than in *indica* genotypes [41]. This is a part of the adaptation process of the rice genotypes to the cool climate [42]. Despite all of this, the ratio of variable fluorescence to maximal chlorophyll fluorescence (Fv/Fm) is a valuable indicator commonly used to assess the tolerance of plants to cold stress [43].

Our objectives were (1) to compare several spectral indices throughout leaf absorbance, reflectance and transmittance with the fast chlorophyll-a fluorescence parameters under cold stress; (2) to develop some new cold sensitive indices to describe the spectral line shape; (3) to find cold-responsive spectral bands; and (4) to determine the differences between tolerant and sensitive rice genotypes.

## 2. Results

### 2.1. The Validation of Handheld Spectrometer Data

**Figure 1:**
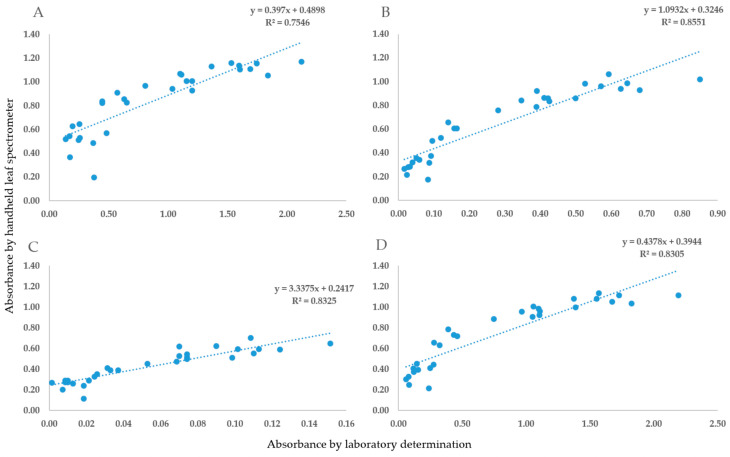
The optical density results of the laboratory determination (*x*-axis) and SpectraVue leaf spectrometer (*y*-axis) measurements at 470 nm (**A**), 647 nm (**B**), 537 nm (**C**) and 663 nm (**D**).

**Figure 2:**
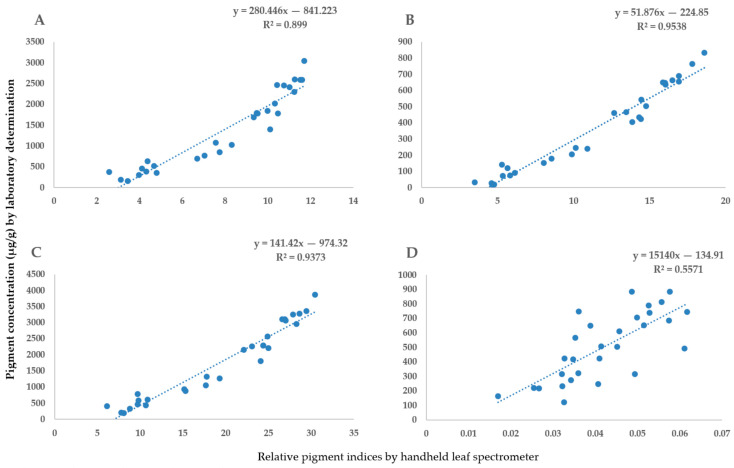
The relationship between the handheld spectrometer indices (CPHLA, CPHLB, CPHLT and CRI 2) and laboratory pigment determination process was as follows: (**A**) CPHLA—chlorophyll-a; (**B**) CPHLB—chlorophyll-b; (**C**) CPHLT—total chlorophyll; (**D**) CRI 2—carotenoid content.

To accurately determine the light absorption parameters measured by the handheld spectrometer, laboratory pigment determination was performed. We compared the light absorbance at four different wavelengths, which is necessary for accurate pigment determination based on Sims and Gamon’s study in 2002 [44]. The optical density data from the two types of measurements (Figure 1), as well as the spectrometry indices and pigment concentrations, were highly correlated (Figure 2).

### 2.2. The Characteristics of the Seasonal Variation and Treatments

We examined sixteen rice genotypes that were exposed to cold stress across two seasons with various temperature ranges. The main effects of the seasonal variation and genotypes were significant. Based on the MANOVA, the parameters of the leaf spectral characteristics and chlorophyll-a fluorescence also significantly depended on the maximum, mean and minimum air temperatures. However, there was no significant correlation among the three calculated temperature parameters. This demonstrates that all three temperature values have the same effect on the variables under consideration (Table 1).

**Figure 3:**
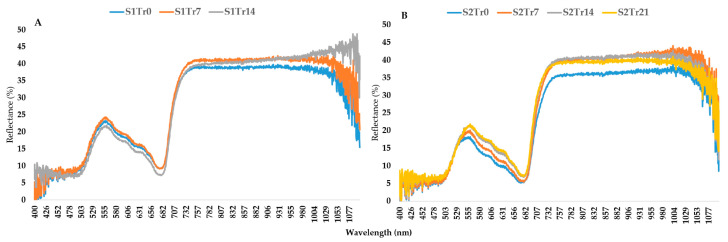
Changes in reflectance patterns under two seasons: (**A**) spring; (**B**) autumn. Every line contains the data for 16 genotypes with 10 replications.

**Figure 4:**
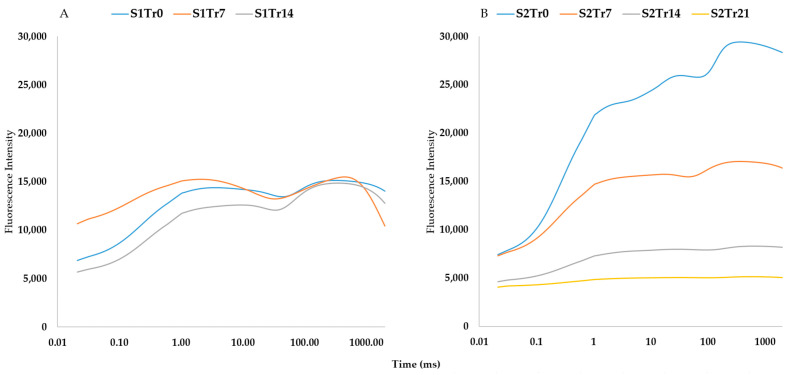
Changes in fast chlorophyll-a fluorescence kinetics (OJIP) patterns under two seasons: (**A**) spring; (**B**) autumn. Every line contains the data for 16 genotypes with 10 replications.

**Figure 5:**
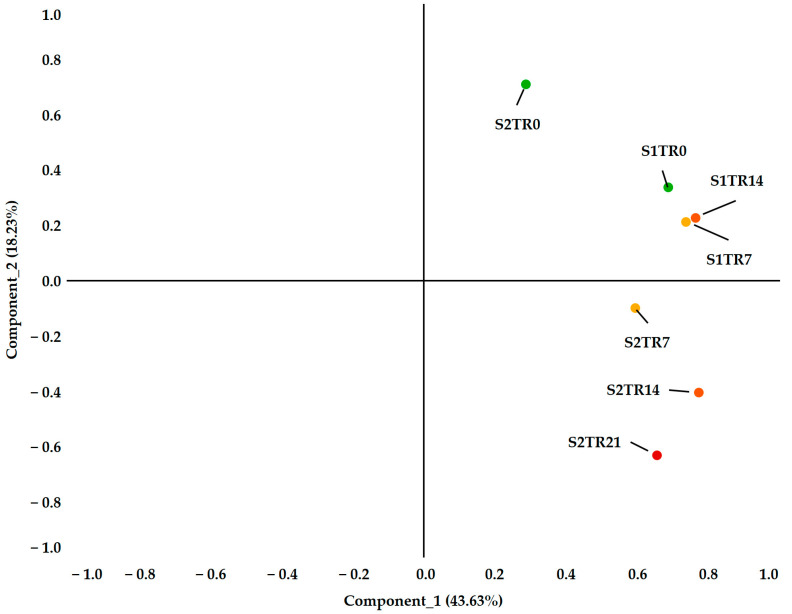
The principal component analysis of all measured spectral and chlorophyll-a fluorescence parameters from 16 genotypes with 10 replications to describe the differences among treatments and seasonal variations.

According to Table 2, the temperature regime was steady during the experimental observations in the spring but significantly decreased in the autumn. This difference between the two seasons is illustrated well by Figure 3 and Figure 4. In comparison to autumn, spring shows a narrower range of mean reflectance values, which is confirmed by the chlorophyll-a fluorescence intensity. However, the second season shows more pronounced differences across the treatments (Figure 4B). These differences were proven by the results of the PCA analysis (Figure 5). It showed high sampling adequacy (KMO = 0.688, sig < 0.001) and two extracted components explained 61.86% of the total variance. The controls of the seasons (S1Tr0 and S2Tr0) reached the highest values for the second component. With the decrease in temperature, the values decreased too. Due to the consistent temperature regime, variations between the three spring treatments (S1) could not be noticed. However, in autumn (S2), the measurements significantly differed from each other. Altogether, the data from the spring observations are useable as repeated information to assess the varieties’ overall performance, while the data from the autumn observations are more suited to calculating the relative decrease in the parameters.

### 2.3. The Wavelengths That Are Affected by Low Temperatures

**Figure 6:**
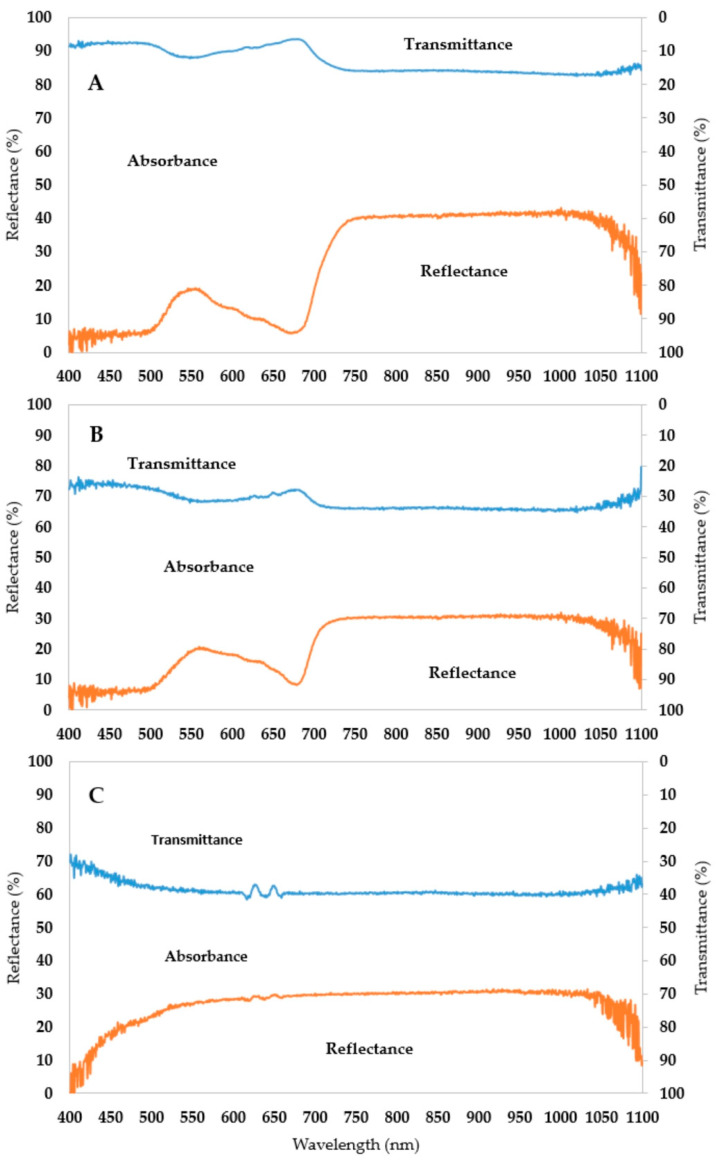
Hyperspectral data for absorbance, reflectance and transmittance in normal (**A**), stressed (**B**) and albino plants (**C**). The normal condition represents the average of the tolerant varieties (M 202, HSC 55 and Kikko) under S2Tr0. The stressed one is showing the sensitive varieties (IR60046-A, IR74371-70-1-1 and N22) at S2Tr21. The albino condition contains all albino seedlings that appear under cold treatment. The orange line represents reflectance, while the blue one is the transmittance.

In normal conditions, plants have a broad absorbance range of visible light spectra (400–700), with the local minimum at 546 nm and the peak at 672 nm. Above 700 nm, the leaf absorbs less light because of the high amount of reflected and transmitted light. Beyond 1050 nm, there is an increasing trend in terms of the absorption intensity (Figure 6A). Under cold stress (Figure 6B), the absorbance rate was narrower than for the control. This was caused by higher amount of transmitted light at all wavelengths and the reflected light in the region of 546–672 nm. Over 700 nm, the reflection typically drops from 40% to 30%. Usually, albino plants exhibit this reflection rate throughout the entire spectrum. The transmittance of the pigmentless seedlings was at a high level throughout the whole spectrum (40%, Figure 6C).

**Figure 7:**
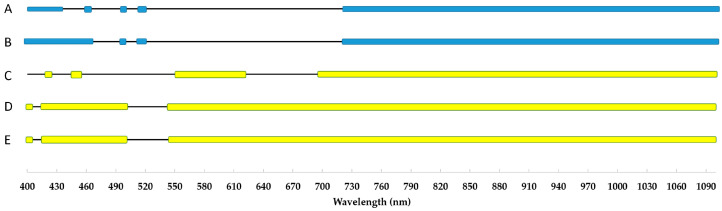
Changes of reflectance patterns under cold stress compared to the control. Each box represents significant changes from the control. The blue boxes indicate the spring measurements, the yellow ones the autumn ones. Every capital letter shows different treatment as follows: A—S1Tr7; B—S1Tr14; C—S2Tr7; D—S2Tr14; E—S2Tr21.

To conduct a more thorough study, we used an ANOVA at a resolution of 5 nm to identify the wavelengths that are impacted by low temperatures when compared to the control. Under cold stress, the reflectance pattern exhibits significant variation (Figure 7), particularly in the 400–700 nm range. One conserved band in this region, between 525 and 535 nm, is completely unaffected by cold for all observations and seasons. The ranges that are very influenced by low temperatures are below 425 nm and above 720 nm. The difference between two growing periods is visible around 510–520 nm and 550–625 nm. These regions are completely opposite to one another (Figure 7).

### 2.4. The Effects of the Genotypes

**Figure 8:**
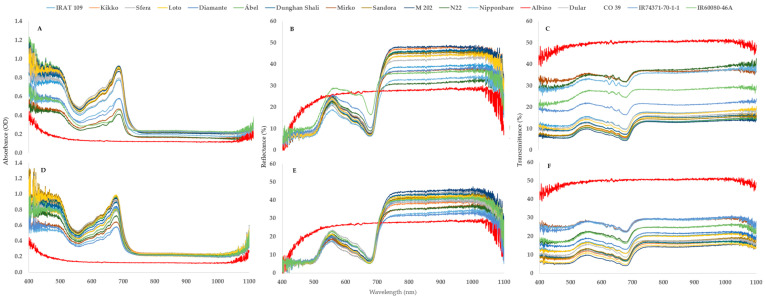
Hyperspectral data of the absorbance (**A**,**D**), reflectance (**B**,**E**) and transmittance (**C**,**F**) in spring (**A**–**C**) and autumn (**D**–**F**). The graphs represent the averages of 3 treatments (S1Tr0, S1Tr7, S1Tr14) (**A**–**C**) or four treatments (S2Tr0, S2Tr7, S2Tr14, S2Tr21) (**D**–**F**). The different colors are equivalent to different varieties.

According to the MANOVA results (Table 1), the effects of the genotypes have a greater impact on the leaf spectral response than seasonal variation. Although Figure 8 supports this idea, there are more differences among the varieties in the spring (Figure 8A–C), particularly between tolerant and sensitive ones, than there are in the autumn.

**Figure 9:**
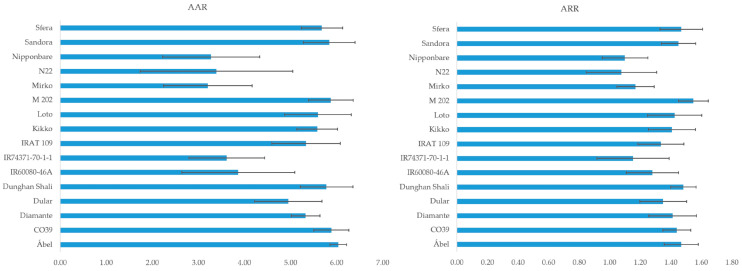
The differences between the genotypes and the albino variety at the absorbance level of 672 nm (albino absorbance ratio—AAR) and the average of the intervals of 800 and 900 nm (albino reflectance ratio—ARR).

In S1 compared to S2, the genotype differences are larger (Figure 8). However, the differences among the genotypes are similar. The highest AAR value was found for the genotype Ábel (6.04), based on the comparison of genotype and albino values (6.04). This value was very stable despite the different temperature regimes across the two seasons. The varieties that had lower AAR values also had high standard deviations. The correlation between the mean of AAR and its SD was linear (y = −0.291x + 2.1468; R^2^ = 0.684). The Mirko variety (3.21) had the lowest AAR. The ratio of the reflectance (RAR) shows that the cold-tolerant M 202 variety reflected 1.55 times more light in the range of 800–900 nm than that of the albino variety. The lowest difference was in case of the N22 variety (Figure 9). The second index that describes the shape of the reflectance curve well is the REIP. The inflexion points of the curve are related to the health status of the plants. A shifted curve to the left is generally observed under cold stress; however, there is a wide range among the genotypes. Under normal conditions (S2Tr0), the range of REIP values was 698.37–710.18 nm, while it was 675.11–709.32 nm under the lowest temperatures (S2Tr21). The highest difference was detected for the N22 variety (24.58 nm) and the lowest was in the case of Nipponbare (0.41 nm). The third area where the effect of the genotype is visible is the increased level of reflectance in the region of the visible range (Figure 8). The closest variety to albino is IR60080-46-A and the farthest is Nipponbare (Figure 8).

**Figure 10:**
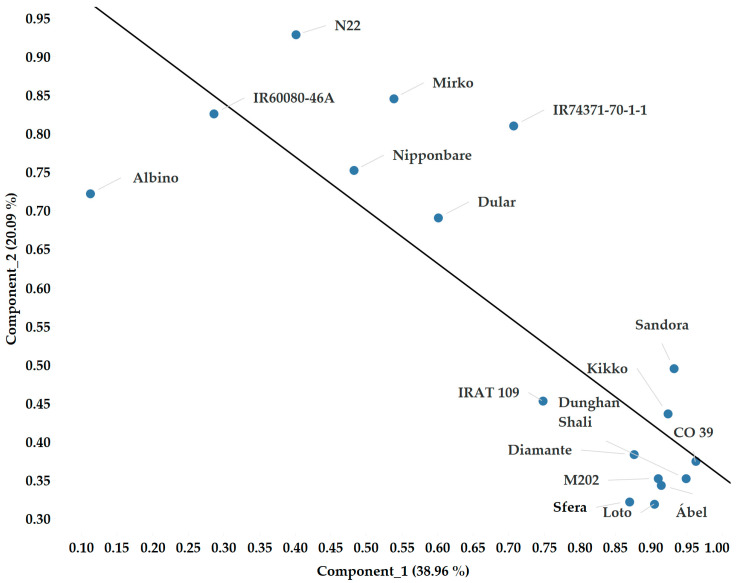
A principal component analysis of all examined indices of leaf spectral distribution and chlorophyll-a fluorescence parameters.

We identified two main components (Figure 10) with the principal component analysis based on all measurements and all spectral and chlorophyll-a fluorescence data across two growing seasons. The assumptions of the PCA showed a high sampling adequacy (KMO = 0.755, sig < 0.001) and correlation among the studied parameters. The two extracted components explained 59.05% of the total variance. The relationship between the two components can be explained by the linear model with high accuracy. The Pearson’s correlation value is −0.831, while the equation of the linear fitting is y = −0.6928x + 1.0471. The standard error of the slope is 0.12, the intercept is 0.09 and their t-values are −5.78 and 11.54. The significance level is <0.0001 and the average squared difference between the observed and predicted values (MSE) is 0.016 (Figure 10). If we exclude the albino plants from the linear fitting, the model tends to be more precise (MSE = 0.01). Based on Figure 10, we can separate the sensitive genotypes (IR60080-46A, N22, Mirko, Nipponbare, IR74371-70-1-1 and Dular) from the tolerant ones (IRAT 109, Diamante, Sandora, Kikko, Dunghan Shali, M 202, Sfera, Loto, CO 39 and Ábel). The performance of IRAT 109 and CO 39 showed unexpected outcomes, since the former genotype belongs to *tropical japonica* and the latter belongs to *indica*.

### 2.5. The Relationship among Spectral Indices and Photosyntetic Activity

**Figure 11:**
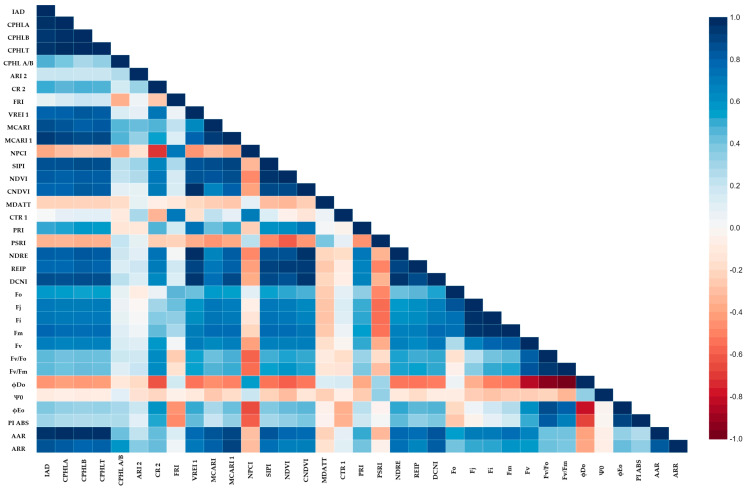
The heat map of Pearson’s correlation coefficients among the studied parameters in spring.

**Figure 12:**
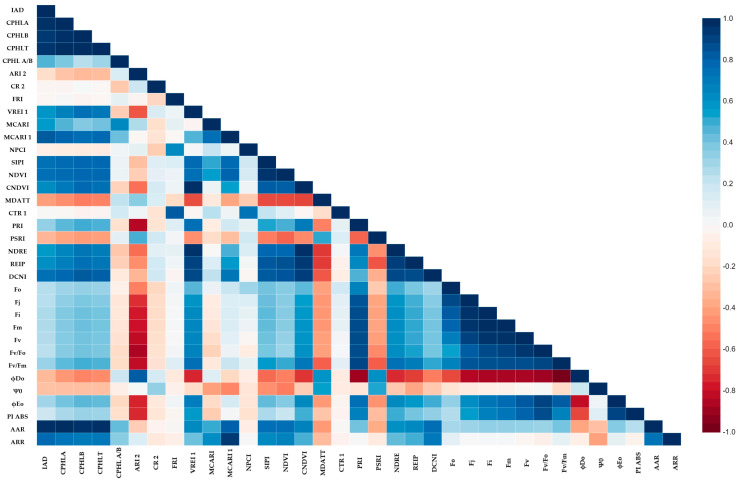
The heat map of Pearson’s correlation coefficients among the studied parameters in autumn.

Based on Pearson’s correlation coefficient, the IAD and AAR indices provided the most accurate estimation of the chlorophyll content (Figure 11 and Figure 12). The PSRI and MDATT have a negative correlation with most of the photosynthetic parameters (Fo, Fi, Fj, Fm, Fv/Fo, Fv/Fm, ϕEo and PI_ABS_) and have a positive correlation with the dissipated energy flux and trapped flux. In contrast, the phytochemical reflectance index (PRI) shows a different pattern of correlation to the photosynthetic parameter. These significant correlations were observed in both spring and autumn. In the spring, there was no chlorophyll-a degradation between the observations when the temperature was low for a longer period of time (Figure 11). However, an increasing CPHL A/B ratio was observed in autumn. The elevated CPHL A/B correlated with lower photosynthetic parameters (Figure 12). Other pigment-related indices, such as ARI 2 and CR 2, also had a significant negative correlation with Fo, Fi, Fj, Fm, Fv/Fo, Fv/Fm, ϕEo and PI_ABS_. In the spring, however, the relationship was the opposite. The flavonols also reacted differently under different temperature regimes. An increased level of flavonols has a negative correlation with the maximum quantum yield of PSII (Fv/Fm), electron transport yield (ϕEo) and overall photosynthesis performance (PI_ABS_) in spring, and there was no significant correlation in autumn. To counter this, the FRI correlates strongly with CTR 1 in both seasons (0.725 **—spring and 0.838 **—autumn).

### 2.6. The Differences between Tolerant and Sensitive Genotypes

**Figure 13:**
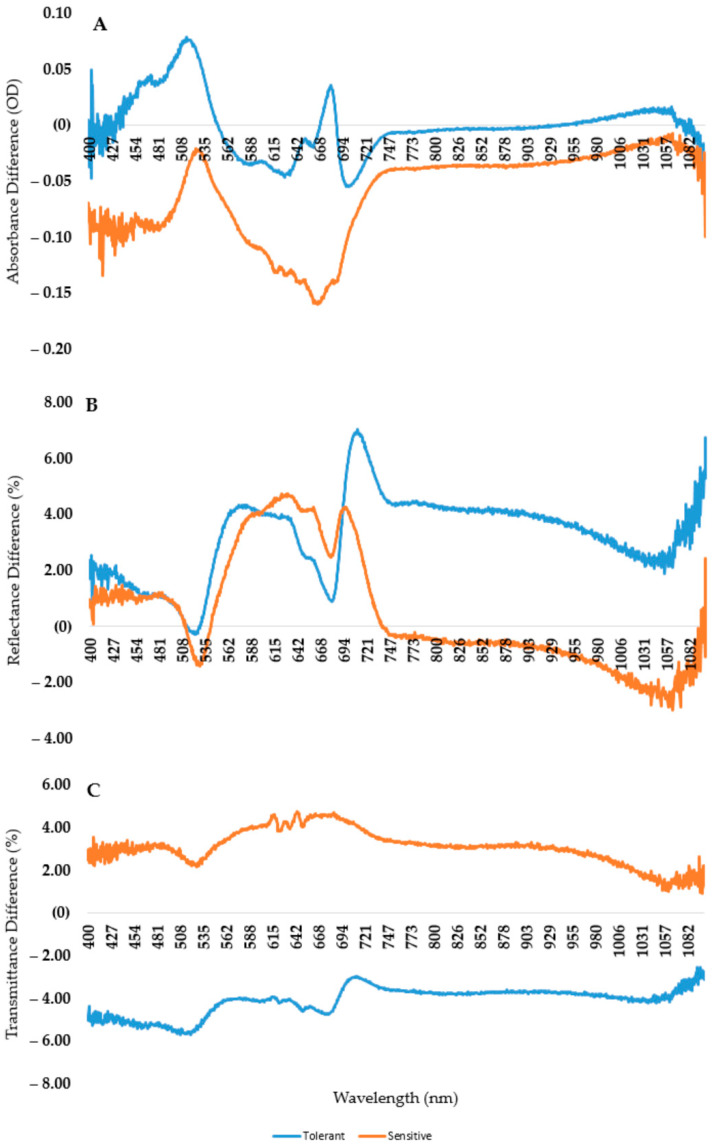
The differences between the control and S2Tr21 variety in terms of absorbance (**A**), reflectance (**B**) and transmittance (**C**).

The variations between genotypes are clearly divided into two groups based on their spectral and chlorophyll-a fluorescence responses, as the principal component analysis (Figure 10) revealed. The highest decrease in mean temperature was observed in the case of the control compared to the S2TR21 variety. We computed a relative change for S2TR21 compared to the control in order to distinguish between tolerant and sensitive varieties (Figure 13). In the case of sensitive varieties, the rate of absorption was reduced across all wavelengths. The shapes of absorbed light curves are similar in the ranges of 400–600 nm and 700–1100 nm but they are shifted. The maximum value was observed in the range of 514–519 nm in the case of the tolerant variety, while it was observed at 525–531 nm in the sensitive one. Additionally, the curves completely diverged between 600 and 700 nm. A second peak was also observed for tolerance lines at 682 nm, but it was absent in the sensitive genotypes. The highest differences were noticed at 677–670 nm. The absorbance rate was stable despite the cold stress at the following wavelengths in the case of the tolerant varieties: 548–553 nm, 667–670 nm, 687–688 nm and 800–950 nm (Figure 13A).

A shift in the curve was also observed in case of the reflectance of the leaves (Figure 13B). In the 523–680 nm range, the curve shifts to the right, while above 680 nm the curve shifts to the left. The first local minimum appears at 523 nm in the case of the tolerant variety and at 528 nm in sensitives ones. There are no differences between the second local minimums of the curve. The minimum wavelength for both tolerant and sensitive varieties was 681 nm. In the tolerant ones, the peak of the reflectance difference was found at 711 nm, whereas in the sensitive ones, it was found at 696 nm. The peak of the reflectance difference was detected at 711 nm in tolerant varieties and at 696 nm in sensitive ones. The tolerant genotypes reflected 4% more light in the infrared band (above 700 nm), whereas the sensitive genotypes reflected 4% less light than the control. Regarding the rates of transmitted light (Figure 13C), the variations between tolerant and sensitive varieties were clearly apparent. Under cold stress, the tolerant plants have around 3–5.7% less light passing through the leaf, whereas sensitive plants have around 1–4% more than the control.

## 3. Discussion

The chilling temperature is an important environmental factor that limits rice production worldwide. Moreover, with the spread of *indica × japonica* hybrids [45], the new varieties are more susceptible, which has a major impact on their uniform, rapid growth in high-altitude and -temperate regions. Therefore, to select suitable genotypes for breeding, early diagnosis of damage induced by low temperatures is required.

We found that the application of different low-temperature regimes is suitable for distinguishing genotypes. In spring, the temperatures were similar across two weeks. The average temperature was close to the point where rice damage begins, at about 15 °C [34,35]. The germination and seedling growth rates of the cold-sensitive cultivars were low, as described in earlier studies [38,46,47,48]. Therefore, this kind of long-term cold stress leads to greater differences among the genotypes than autumn screening (Figure 3 and Figure 4). The benefit of sowing in autumn is the higher temperature, which provides uniform seedling establishment across different varietal groups. Because of the gradual temperature decrease, the first appearance of low temperatures can be monitored using a spectrometer and fluorimeter.

Based on the ANOVA with the resolution of 5 nm, we detected a wide range of wavelengths sensitive to cold stress. In terms of long-term stress (S1Tr0, S1Tr7, S1Tr14), the 470–495 nm and 525–715 nm ranges are totally unaffected by cold. The 405–440, 460–465, 500, 515–520 and 720–1100 nm bands are the most sensitive to chilling temperatures because these wavelengths changed for both treatments (S1Tr7, S1Tr14). The rest of the wavelengths (400, 445–455, 505–510 nm) changed only for the latest measurement (S1Tr14). The stability of the 525–535 nm range and the sensitivity above 720 nm were also confirmed by the autumn screening (Figure 7). Beside the sensitivity of the near-infrared region, the first sign of cold damage was in the 425 nm, 450–455 nm and 550–620 nm bands (Figure 7). The 425 nm and 550 nm bands are the absorption peaks of the carotenoids [49]. The sensitivity at 550 nm is also confirmed as being arsenic stress [19] and several other stresses such as competition, ozone stress and senescence [16]. Our findings supported those of Obeidat et al. (2018), who found a significant temperature effect in the 500–675 nm range and attributed it to variations in chlorophyll content [14]. The 525 nm wavelength is traditionally used to measure anthocyanin in solutions [36], although in intact leaves the 550 nm band correlated well with the analytical determination [50]. In addition, it has been reported with a wide range of plant species that the high photon fluxes near 530 nm induce small reversible changes in reflectance, which can be attributed to xanthophyll conversion of the violaxanthin cycle [12].

Among the three factors (temperature, seasonal variation and genotype), the genotypes had the highest impact on the leaf spectral and chlorophyll-a fluorescence parameters (Table 1), which is consistent with the results of an examination of cold stress in maize [14]. The most remarkable differences were observed at 550–700 nm in terms of absorbance and above 700 nm in terms of reflectance among the genotypes (Figure 8). We developed two new indices (AAR and ARR), which are linked to chlorophyll degradation, in addition to the well-known indices, IAD and REIP, which correlated with the chlorophyll content [17] and stress level [23,24]. We compared the current values for absorbance and reflectance to the albino variety. The AAR index was calculated for the absorbance peak at 672 nm in contrast to Liu et al. **[51]**, who observed an absorbance maximum at 669 nm. Based on two seasons, the closest genotype to albino was Mirko and the outmost was Ábel. This variety surpasses one of the cold-tolerant standards, M 202 [35,36]. However, Nipponbare, which was reported previously as being a cold-tolerant variety [36]**,** had a low AAR index, along with other sensitive varieties (N22, IR60080-46A and IR74371-70-1-1). A similar conclusion can be made for the ARR. A higher reflectance rate was observed in the case of the tolerant variety than its sensitive ones. This complements the findings from studies using heat [52], cold [14] and heavy metal stresses [19]. However, Katsoulas et al. reported that the reflectance of stressed plants was increased in the near-infrared region [53].

The AAR index had positive correlations with chlorophyll (CPHLA, CPHLB, CPHLT, MCARI, MCARI1 AND SIPI), nitrogen (NDRE, DCNI AND REIP) and fluorescence (Fo, Fj, Fi, Fm, Fv, Fv/Fm) parameters across two seasons (Figure 11 and Figure 12). The co-movement of chlorophyll and nitrogen has been reported by several authors [8,53]. This may be because the chilling temperature not only inhibits the synthetization of chlorophyll and starts its degradation [37] but also limits nitrogen uptake [38]. Negative correlations were observed with NPCI, PSRI, ϕDo and Ψ0. The NPCI indicates the carotenoid/chlorophyll ratio, which is higher under cold stress, especially in nitrogen-limiting environments [54]. A similar observation can be made for the AAR, but it had lower correlations with the fluorescence parameters, except ϕDo. The plants with a greater distance from the albino variety in terms of the reflectance rate in the infrared region can absorb high-frequency light, which would cause irreversible denaturation of the protein [55].

There are several evaluation methods used to perform a comparative statistical study to find the spectral bands with high correlations to the variables. Several papers reported that the evaluation of spectral band results allows a better comparison with a principal component analysis [7,8,18,56]. Using this method, we examined the entire dataset, which included data from 16 different genotypes, two seasons and both spectral and fluorescence data (Figure 10). The outcome revealed two major groups. A comparison between the two groups was also carried out. We found that the maximum absorbance value was in the 514–519 nm range in the case of the tolerant variety, while it was at 525–531 nm in the sensitive one. After the local minimum at around 630 nm, the absorbance difference was increased in the case of the tolerant variety and decreased in the sensitive ones. Therefore, the greatest difference was observed at 682 nm (Figure 13A). Similar shifted curves in reflectance were reported by Ishikawa et al. in 2015 and Obeidat et al. in 2018 [14,15]. However, in our results, we detected two methods of shifting in the case of reflectance. In the range of 523–680 nm, the curve shifts to the right, while above 680 nm, the curve shifts to the left. Moreover, the peak of the reflectance difference was detected at 711 nm in the tolerant varieties and at 696 nm in the sensitive ones.

## 4. Materials and Methods

The plant materials were selected from the Rice Variety Collection maintained by the MATE ÖVKI Galambos Rice Research Station (46°52017.500 N 20°31037.500 E, Szarvas, Hungary). The selection criteria for the genotypes were that the varieties represent a wide range of cold tolerances and varietal groups (Table 3).

**Figure 14:**
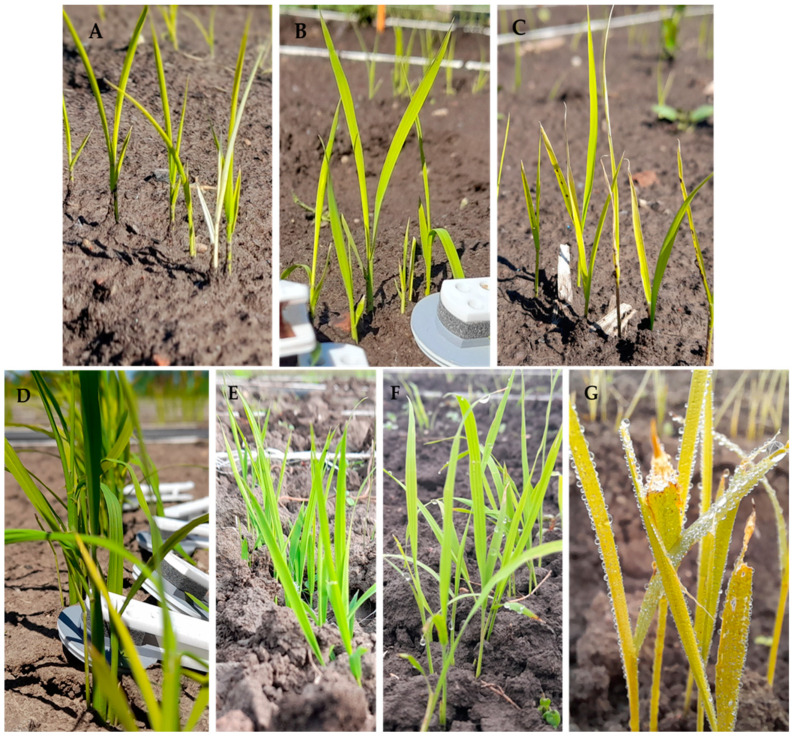
The plant population for the experiment in two seasons (spring (**A**–**C**) and autumn (**D**–**G**). The letters indicate the treatments as follows: (**A**) S1Tr0; (**B**) S1Tr7; (**C**) S1Tr14; (**D**) S2Tr0; (**E**) S2Tr7; (**F**) S2Tr14; (**G**) S2Tr21. (**A**) This image also indicates the reference albino seedling. (**B**,**D**) These images demonstrate the clips of the chlorophyll-a fluorescence meter.

Each genotype was sown in a 1 m row in a paddy field. The plants were grown in two seasons (spring and autumn) of 2021. Usually, the cold weather under germination phase appears in the temperate rice-growing area in spring. Due to the effect of low temperatures under early developmental phases, the seedlings are not uniform and severe damage is suffered. This could happen in the cold-sensitive varieties, especially in *indica* plants. In order to avoid non-uniform seedlings, we applied a second repetition in autumn. In this case, the soil temperature was higher and the seedlings were well-developed and uniform. The conditions of the seedlings, the differences between seasons and the degree of cold stress can be clearly seen in Figure 14.

The temperature conditions during the spring and autumn for the measurements are shown in Table 2. The meteorological data were provided by Boreas Agromet-Solar automatic meteorological stations and the soil temperature data were provided by DeltaT SM150T (Cambridge, UK) soil moisture and temperature probes with GP1 data loggers.

The measurements were taken at the 2–3 leaf stage by a Spectavue leaf spectrometer (Cid-BioScience, Camas, WA, USA) and PAR FluorPen instrument (PSI, Drásov, Czech Republic) with ten biological replications. The chlorophyll fluorescence measurements were performed after dark adaptation for 20 min. A saturating pulse induced to measure the maximum chlorophyll fluorescence was applied at 2000 µmol·m^2^·s**^−^**^1^. The following indices were calculated based on leaf absorbance, reflectance and rapid chlorophyll kinetics and the OJIP test (Table 4).

The chlorophyll-a (CPHLA), chlorophyll-b (CPHLB), total chlorophyll (CPHLT) and carotenoid (CRI 2) indices calculated by handheld leaf spectrometer were validated via laboratory pigment determination [44]. We chose thirty plants with different CPHLT values (5–30) to determine the pigment concentration. The absorbance of the pigment solution was measured by a DR 4000 spectrophotometer (Hach Company, Loveland, CO, USA) and the following equations were used for determination of the pigments:

$${\mathrm{Chl}}_{a}=0.01373A_{663}-0.000897A_{537}-0.003046A_{647}$$

$${\mathrm{Chl}}_{b}=0.02405A_{647}-0.004305A_{537}-0.005507A_{663}$$

$$\mathrm{Carotenoids}=(A_{470}-\left(17.1\times \left({\mathrm{Chl}}_{a}+{\mathrm{Chl}}_{b}\right)-9.479\times \;\mathrm{Anthocyanin}\right))119.26$$

A statistical analysis was performed (MANOVA) first as an overall test to determine the effect sizes of the seasonal variation, genotype and temperature. Second, we reduced the resolution of the hyperspectral data from 1 nm to 5nm. Using every 5nm band, we ran an ANOVA to determine which wavelengths are affected by temperature. To illustrate the differences among genotypes, indices and seasonal variation, we used a principal component analysis. For the analysis, we used factors above 1.0 eigenvalue. Three extracted components explained high percentages of the total variance (70–80%). To test the correlations among leaf spectral and chlorophyll-a fluorescence parameters, we used Pearson’s correlation. To simplify the hyperspectral data, a comparison to the albino plants was carried out. The statistical analysis was carried out using IBM SPSS v25. The visualization of the data was performed using Microsoft Excel 2016, DisplayR and Tableau v2020.1.

## 5. Conclusions

In this paper, we analyzed the changes in leaf spectral and chlorophyll-a fluorescence parameters under natural cold stress in rice. We made a comparison to spectral line shape of the albino plant, and we identified the characteristic points of the curves of absorbance, reflectance and transmittance. The calculated vegetation indices were validated by chlorophyll-a fluorescence parameters. Based on a principal component analysis, we identified the tolerant and sensitive genotypes. We defined the cold-responsive wavelength and which ones differed between tolerant and sensitive varieties. The following conclusions can be drawn:

(1) The spring testing environment is more suitable for marking differences among genotypes based on their long-term stress responses. On the other hand, in autumn, the susceptibility of each variety to chlorophyll degradation is higher. Thus, the extent of the decline can be determined better;
(2) The spectral pattern of an albino leaf can be characterized with a narrow absorbance range. The mean amount of the reflected light is around 30% and the transmitted amount is 40% throughout the whole spectrum;
(3) Albino plants are the theoretical endpoint of chlorophyll degradation, so the new difference indices (AAR and ARR) are suitable for better describing the shape of the curve and the extent of chlorophyll degradation;
(4) The most stable wavelength range to cold stress was 525–535 nm, while the most sensitive was above 700 nm in the reflectance curve;
(5) Almost all wavelengths outside the 525–535 nm range are suitable for differentiating between tolerant and sensitive varieties based on the control and cold-treated spectrograph difference.

## Figures and Tables

**Table 1:** MANOVA outputs of seasonal variation, genotype and temperature. The values are calculated by Wilks’ lambda.

	Value	F	Hypothesis df	Error df	Sig.
Genotype	0.000	11.733	512.000	6921.549	0.000
Seasonal variation	0.094	144.406	32.000	478.000	0.000
T_max_	0.000	64.409	192.000	2987.519	0.000
T_mean_	0.000	64.409	192.000	2987.519	0.000
T_min_	0.000	64.409	192.000	2987.519	0.000

**Table 2:** Seven-day averages of air and soil temperatures during the experiment.

				Air Temperature		
	Date	Day	Code	T_max_	T_mean_	T_min_
Spring	19 May 2021	0	S1Tr0	19.27	13.99	9.17
	26 May 2021	7	S1Tr7	21.27	15.20	9.59
	2 June 2021	14	S1Tr14	21.24	15.13	9.20
Autumn	27 September 2021	0	S2Tr0	24.16	18.19	9.83
	4 October 2021	7	S2Tr7	20.16	15.85	9.81
	11 October 2021	14	S2Tr14	16.60	12.53	7.18
	18 October 2021	21	S2Tr21	13.93	8.76	1.48

**Table 3:** List of genotypes, countries of origin and varietal groups.

Name	Country of Origin	Varietal Group
Ábel	Hungary	*temperate japonica*
Dunghan Shali	Hungary	*temperate japonica*
Sandora (HSC 55)	Hungary	*temperate japonica*
Kikko	Italy	*temperate japonica*
Sfera	Italy	*temperate japonica*
Loto	Italy	*temperate japonica*
Diamante	Chile	*temperate japonica*
M 202	U.S.A.	*temperate japonica*
Nipponbare	Japan	*temperate japonica*
Mirko	Italy	*tropical japonica*
IRAT 109	Ivory Coast	*tropical japonica*
IR60080-46A	Philippines	*tropical japonica*
N22	India	*aus*
Dular	India	*aus*
CO 39	Philippines	*indica*
IR74371-70-1-1	Philippines	*indica*

**Table 4:** Vegetation indices and chlorophyll fluorescence parameters used for studying the cold tolerance and their equations and references.

Index	Formulation	Reference
Absorbance Difference Index (I_AD_)	A670 − A720	[17]
Chlorophyll A (CPHLA)	(12.7 × A663) − (2.59 × A645)	[57]
Chlorophyll B (CPHLB)	(22.9 × A645) − (4.7 × A663)	[57]
Chlorophyll TOTAL (CPHLT)	(8.2 × A663) + (20.2 × A645)	[57]
Chlorophyll a/b Ratio (CPHLA/CPHLB)	CPHLA/CPHLB	[42]
Anthocyanin Reflectance Index 2 (ARI 2)	R800 × (1/R550) − (1/R700)	[50]
Carotenoid Reflectance Index 2 (CRI 2)	(1/R510) − (1/R700)	[12]
Flavonols Reflectance Index (FRI)	(1/R410 − 1/R460) × R800	[13]
Vogelmann Index (VREI 1)	R740/R720	[58]
Modified Chlorophyll Absorption Ratio Index (MCARI)	((R700 − R670) − 0.2 × (R700 − R550)) × (R700/R670)	[59]
Modified Chlorophyll Absorption Ratio Index 1 (MCARI1)	1.2 × (2.5 × (R800 − R670) − 1.3 × (R800 − R550))	[60]
Normalized Pigment Chlorophyll Index (NPCI)	(R680 − R430)/(R680 + R430)	[54]
Structure Intensive Pigment Index (SIPI)	(R800 − R445)/(R800 + R680)	[61]
Normalized Difference Vegetation Index (NDVI)	(R800 − R680)/(R800 + R680)	[60]
Red Edge NDVI (RENDVI)	(R750 − R705)/(R750 + R705)	[62]
Modified DATT Index (MDATT)	(R719 − R726)/(R719 − R743)	[63]
Carter Index (CTR 1)	R695/R420	[10]
Photochemical Reflectance Index (PRI)	(R531 − R570)/(R531 − R570)	[32]
Plant Senescence Reflectance Index (PSRI)	(R680 − R500)/R750	[62]
Normalized Difference Red Edge (NDRE)	(R790 − R720)/(R790 + R720)	[64]
Red Edge Inflection Point (REIP)	700 + 40 × (((R670 + R870)/2 − R700)/(R740 − R700))	[65]
Double-peak Canopy Nitrogen Index (DCNI)	(R720 − R700)/((R700 − R670)/(R720 − R670 + 0.03))	[66]
Albino Absorbance Ratio (AAR)	A672 of genotype/A672 of albino	This paper
Albino Reflectance Ratio (ARR)	Average of R800:R900/Average of R800:R900 of the albino	This paper
Fluorescence Intensity at 50 µs (Fo)		[67]
Fluorescence Intensity at 2 ms (Fj)		[67]
Fluorescence Intensity at 30 ms (Fi)		[67]
Maximal Fluorescence Intensity (Fm)		[67]
Variable Chlorophyll Fluorescence (Fv)	Fv = (Fm − Fo)	[67]
Maximum Quantum Yield of PSII (Fv/Fm)	Fv/Fm	[67]
Trapped Flux (Ψ0)	Psi_0 = 1 − Vj	[67]
(Fv/Fo)	(Fv/Fo)	[29]
Electron Transport Quantum Yield (ϕEo)	ϕEo = (1 − Fo/Fm)·Ψ0	[29]
Thermal Dissipation Quantum Yield (ϕDo)	(Fo/Fm)	[29]
Performance Index (PI_ABS_)	(RC/ABS) × (ϕPo/(1 − ϕPo)) × (ψ0/(1 − ψ0))	[29]

## Data Availability

All data used in this manuscript are presented in the manuscript.
